# Effects of Calcination Temperature and Acid-Base Properties on Mixed Potential Ammonia Sensors Modified by Metal Oxides

**DOI:** 10.3390/s110202155

**Published:** 2011-02-11

**Authors:** Atsushi Satsuma, Makoto Katagiri, Shiro Kakimoto, Satoshi Sugaya, Kenichi Shimizu

**Affiliations:** 1 Graduate School of Engineering, Nagoya University, Nagoya 464-8603, Japan; E-Mails: katagiri.makoto@g.mbox.nagoya-u.ac.jp (M.K.); kshimizu@cat.hokudai.ac.jp (K.S.); 2 Engineering R&D Group, R&D Center, NGK Spark Plug Co. Ltd., Komaki 485-8510, Japan; E-Mails: s-kakimoto@mg.ngkntk.co.jp (S.K.); s-sugaya@mg.ngkntk.co.jp (S.S.); 3 Hokkaido University Catalysis Research Center, Sapporo 001-0021, Japan

**Keywords:** ammonia sensor, metal oxide, YSZ, acidity, melting point

## Abstract

Mixed potential sensors were fabriated using yttria-stabilized zirconia (YSZ) as a solid electrolyte and a mixture of Au and various metal oxides as a sensing electrode. The effects of calcination temperature ranging from 600 to 1,000 °C and acid-base properties of the metal oxides on the sensing properties were examined. The selective sensing of ammonia was achieved by modification of the sensing electrode using MoO_3_, Bi_2_O_3_ and V_2_O_5_, while the use of WO_3,_ Nb_2_O_5_ and MgO was not effective. The melting points of the former group were below 820 °C, while those of the latter group were higher than 1,000 °C. Among the former group, the selective sensing of ammonia was strongly dependent on the calcination temperature, which was optimum around melting point of the corresponding metal oxides. The good spreading of the metal oxides on the electrode is suggested to be one of the important factors. In the former group, the relative response of ammonia to propene was in the order of MoO_3_ > Bi_2_O_3_ > V_2_O_5_, which agreed well with the acidity of the metal oxides. The importance of the acidic properties of metal oxides for ammonia sensing was clarified.

## 1. Introduction

The Uera-SCR (Selective Catalytic Reduction) technique is known to be an effective technology for the removal of nitrogen oxide (NOx) emissions from heavy-duty diesel engine cars [1–4]. In this system, an aqueous solution of urea is injected into a catalytic converter, hydroxylation of urea in the converter results in the formation of NH_3_, and the thus formed NH_3_ then successfully reduces NOx to N_2_ over Fe-zeolite or vanadium-based catalysts in a wide range of temperatures. The urea-SCR system has been already put into practical application, however, monitoring of the NH_3_ concentration in the catalytic converter is required to achieve proper operation of a urea-SCR system. For the practical application of the ammonia sensors to automobile exhausts, sufficient response altitude and cross-sensitivity, quick response, and tolerance to high temperatures under hydrothermal conditions are required.

Various types of ammonia sensors have been proposed [5,6]. The ammonia sensors using surface proton-conducting metal oxides, such as zeolites [7,8] and WO_3_/ZrO_2_ [9,10], show excellent cross-sensitivity to NH_3_ in the presence of various interfering gases, such as hydrocarbons, CO, and NOx. However, these materials have high surface area, and consequently they should have low thermal stability. Semiconductors of n-type metal oxides such as WO_3_ [11], MoO_3_ [12–15], V_2_O_5_ [16,17], SnO_2_ [18,19], TiO_2_ [20], In_2_O_3_ [21–23] and Ru/ZnO [24] have high hydrothermal stability, and have been extensively investigated as sensing materials. They usually act at lower temperatures (below 300 °C) than those needed in the automobile industry, but show low cross-sensitivity to NH_3_ in the presence of various interfering gases. Consequently, it is highly desirable to develop thermally stable ammonia sensors which show high cross-sensitivity to NH_3_ at high temperatures.

Mixed potential sensors are thought to be one of the promising technologies for this purpose because they are used at high temperatures around 500–600 °C. They are usually applied to sensors for CO and hydrocarbons [25–38], however, selective ammonia sensors can be designed by selection of appropriate sensing materials. Wang *et al*. examined various metals and metal oxides as sensing electrodes for ammonia sensors, and demonstrated that V_2_O_5_, BiVO_4_, MoO_3_, and WO_3_ are all effective for the sensing of NH_3_ [39]. Especially, BiVO_4_ showed the best output voltage in the presence of NH_3_, which was far higher than those of CO, C_3_H_6_, and NO. Schönauer and co-workers developed a novel selective ammonia sensor based on the mixed potential effect using a porous V_2_O_5_-WO_3_-TiO_2_-based SCR catalyst as a sensing material [40]. The proposed sensor showed good cross-sensitivity to NH_3_, and they demonstrated that the sensor can detect very small NH_3_ slips at the downstream of a real SCR catalyst. Elumalai *et al*. fabricated a planar mixed-potential-type sensor using a YSZ electrolyte and NiO/Au sensing electrode [41]. The sensor exhibited good sensitivity and cross-sensitivity to NH_3_ at 800 °C under wet conditions, *i.e*., the emf response to 100 ppm NH_3_ was about −34 mV, while the cross-sensitivities to the other examined gases were about ±5 mV or negligible. Hibino *et al*. prepared a proton-conducting thin Zr_1−x_Y_x_P_2_O_7_ film on a YSZ substrate by reacting with liquid H_3_PO_4_ [42]. This sensor yielded a remarkably sensitive and selective response to low concentrations of NH_3_. Their approach suggests a strong contribution of acidity to selective NH_3_ detection.

It can be expected that the acid properties of the sensing material is one of the important factors for better cross-sensitivity because NH_3_ is a basic molecule, while the other infering gasses, CO, HC, and NOx, are not. However, the effects of the acid-base properties of sensing materials have not been clarified. The aim of this study was to obtain knowledge for the design of a metal oxide-modified mixed potential ammonia sensor. From the relationships between sensing properties and character of the metal oxides, the important factors for the selective sensing of NH_3_ are clarified.

## 2. Experimental Section

### 2.1. Materials Synthesis and Sensor Setup

MnO_2_, MoO_3_, Bi_2_O_3_, WO_3_, Nb_2_O_5_, and MgO (99% purity) were purchased from Kishida Chemical Co., Ltd. V_2_O_5_ (99% purity) was purchased from Mituswa Chemical Co., Ltd. BiVO_4_ was prepared by milling V_2_O_5_ and Bi_2_O_3_ for 24 h, followed by calcination of the mixture at 900 °C.

**Figure 1.**
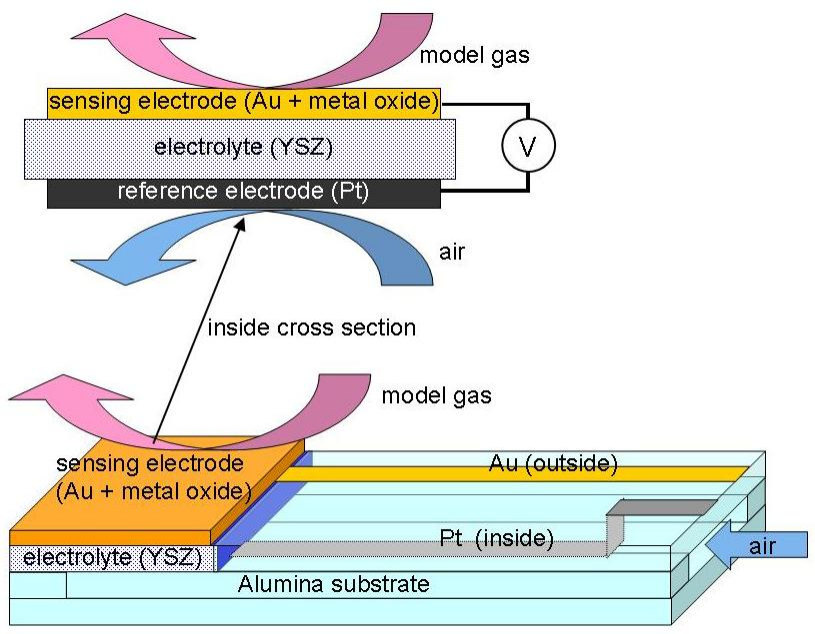
Schematic representation of the sensor examined in the present study.

The schematic structure of the two-chamber cell constructed with a YSZ solid electrolyte on an alumina substrate is illustrated in Figure 1. One side of the YSZ solid electrolyte covered by a Pt electrode is exposed to the inside chamber with outside air. Another side of the YSZ solid electrolyte is covered with a mixture of Au and metal oxide thick film as a sensing material prepared by the screen-printing technique in the same manner reported previously [9,10,17]. For the preparation of electrodes, screen printable pastes were produced by mixing gold paste (purchased from Daiken Chemical Co., Ltd., Au100-1) with 10 wt% of the metal oxide powders. The sensor was calcined in air for 5 h at 650–1,000 °C depending on the metal oxide use, and assembled in a stainless case. Thickness of the film after the calcination step was *ca*. 10–20 μm. The name of each sensor electrode is abbreviated as the name of the metal oxide and calcination temperature in °C, for example, Bi_2_O_3_(850).

### 2.2. Gas Sensor Measurements

Sensing characteristics were evaluated by using a conventional gas-flow apparatus equipped with a furnace operating at 600 °C. The stainless flow cell was heated at operating temperatures. The reference electrode (cathode) was exposed to air, and the sensing electrode (anode) was exposed to a flow of mixture gas. The composition of the base gas is 10% O_2_, 3% H_2_O, and N_2_ as a balance gas at a flow rate of 150 cm^3^ min^−1^. The standard concentrations of NH_3_ and C_3_H_6_ are 500 ppm. The electromotive forces (EMF) value of the cell was measured by an electrometer (Hokuto Denko HZ-5000).

## 3. Results and Discussion

### 3.1. Effect of Calcination

**Figure 2.**
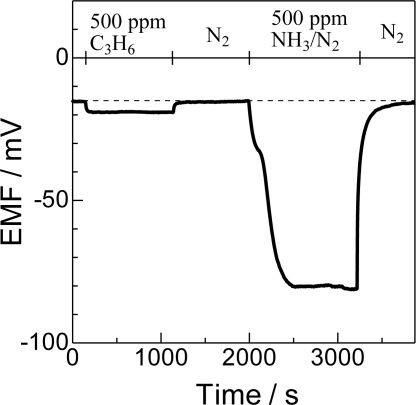
Response of a Bi_2_O_3_(850) sensing electrode to 500 ppm C_3_H_6_ and NH_3_ at 600 °C.

Figure 2 shows an example of transient response of Bi_2_O_3_(850) to the injection of ammonia and C_3_H_6_. Due to the difference in the oxygen concentration (20% at the reference electrode and 10% at the sensing electrode), the EMF of the sensor electrode is −15.5 mV in the absence of reducing gases. In the presence of 500 ppm C_3_H_6_, EMF decreased to −19.1 mV. The difference in EMF between those in the presence and absence of a probe molecule is determined as ΔEMF, 3.6 mV in this case, and used as an indicator of response height. On the other hand, the ΔEMF in a flow of the same amount of NH_3_ was −68.2 mV, which is one order higher than that in C_3_H_6_, indicating the very high selective sensing of the Bi_2_O_3_(850) electrode. After the evacuation of NH_3_, the EMF value quickly recovered to the original level. The ΔEMF of NH_3_ relative to that of the same concentration of C_3_H_6_ (ΔEMF_NH3_/ΔEMF_C3H6_) is used as a measure of cross-sensitivity.

**Figure 3.**
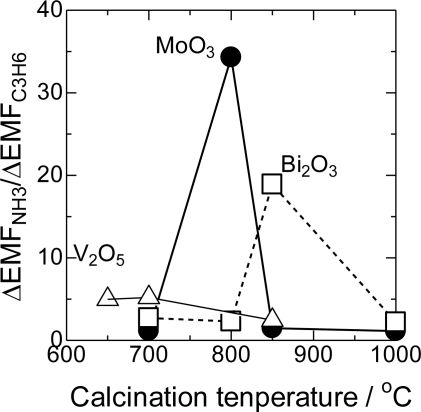
The relative ΔEMF as a function of calcination temperature of sensing electrodes.

The cross-sensitivity strongly depended on the calcination temperatures of the electrodes. In Figure 3, the ΔEMF_NH3_/ΔEMF_C3H6_ ratios of the selected sensors as a function of the calcination temperature are shown. V_2_O_5_, MoO_3_, and Bi_2_O_3_ showed the maximum ΔEMF_NH3_/ΔEMF_C3H6_ at 700, 800, and 850 °C, respectively. Since the melting points of these metal oxides are 695, 790, and 820 °C, the maximum cross-sensitivity was observed around their melting points.

Various kinds of the sensing electrodes listed in Table 1were prepared using the corresponding metal oxides. Due to the strong dependence of the cross-sensitivity on calcination temperatures as shown in Figure 3, the sensing performances were compared after the calcination at the optimum temperatures ranging from 700 to 1,000 °C. For WO_3_(1000), Nb_2_O_5_(1000), and MgO(1000) electrodes, the ΔEMF_NH3_/ΔEMF_C3H6_ ratios were comparable to that of the electrode without modification (Au only). This implies a negligible chemical interaction between Au and metal oxides, which results in the ΔEMF_NH3_/ΔEMF_C3H6_ ratio of around unity and thus, non-selective NH_3_ detection. Melting points of these metal oxides were higher than the calcination temperature of 1,000 °C.

On the other hand, very high ΔEMF_NH3_ and low ΔEMF_C3H6_ were obtained when MoO_3_ was used as a sensing material. As a result, the ΔEMF_NH3_/ΔEMF_C3H6_ ratio was the highest for MoO_3_(800). MnO_2_, V_2_O_5_, and Bi_2_O_3_ also showed high ΔEMF_NH3_/ΔEMF_C3H6_ ratios and sufficient ΔEMF values in the presence of NH_3_. In these metal oxides melting points’ were below 820 °C, and the calcination temperatures were optimized around their melting points. The results show that the melting point of the sensing material is one of the important factors for the preparation of the selective sensors.

The maximum cross-sensitivity at around the melting points of the metal oxides suggests that the good mixing of the metal oxides, Au electrode, and YSZ is essential for selective NH_3_ detection. It can be speculated that the acidic metal oxides are well spread on Au and YSZ surface and achieved the successful modification of Au and YSZ. Above the melting points, too much spreading of the metal oxide results in the undesired covering of Au and YSZ electrode. The strong dependence of the selective sensing of NH_3_ on the calcination temperature suggests that the spreading of metal oxide is important for the successful preparation of the sensor. As shown in Table 1, MoO_3_(850) showed the highest response altitude and ΔEMF_NH3_/ΔEMF_C3H6_ ratio. However, owing to sublimation of MoO_3_ at 1,155 °C [43], MoO_3_ is not suitable for the application of ammonia sensing in automobile exhaust systems. Therefore, further investigations have been done by using Bi_2_O_3_(850) which showed the second highest ΔEMF_NH3_/ΔEMF_C3H6_ and sufficient response altitude.

### 3.2. Effect of Acid-Base Property

**Figure 4.**
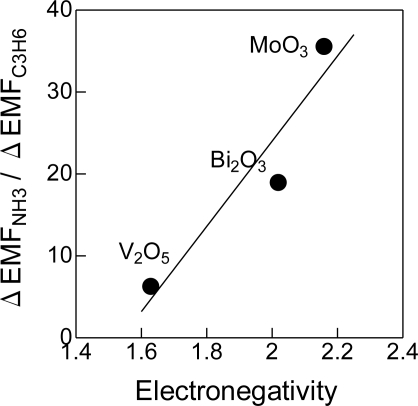
The relative ΔEMF as a function of electronegativity of added metal oxides.

Figure 4 shows ΔEMF_NH3_/ΔEMF_C3H6_ ratio of MoO_3_(800), Bi_2_O_3_(850) and V_2_O_5_(700) as a function of electronegativity of the metals in the metal oxides. As we have reported in a previous paper [17], the electronegativity can be used as an indicator of the acid-base properties of metal oxides: the higher electronegativity implies higher acidity of the oxides. Clearly, the ΔEMF_NH3_/ΔEMF_C3H6_ ratio increased with the electronegativity. This correlation shows that the acidity of metal oxide is very essential for the cross-sensitivity to NH_3_.

**Figure 5.**
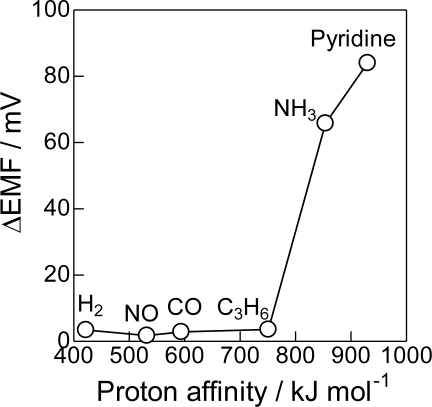
The ΔEMF in the presence of 500 ppm of various gases as a function of the proton affinity.

The importance of acid-base properties also can be seen in the effect of the acid-base properties of probe molecules shown in Figure 5. The proton affinity is a good indicator for the acid-base properties of molecules [44]. The Bi_2_O_3_(850) electrode was not sensitive to H_2_, NO, CO, C_3_H_6_ having lower proton affinity. However, ΔEMF became very high to NH_3_ and pyridine which have high proton affinity, *i.e*., are highly basic molecules. These results clearly indicate that the sensing response of the prepared electrodes strongly depends on the acid-base interaction of the sensing electrodes and probe molecules. A highly acidic property is required for the selective detection of NH_3_.

### 3.3. Performance of Bi_2_O_3_(850) as an Ammonia Sensor

**Figure 6.**
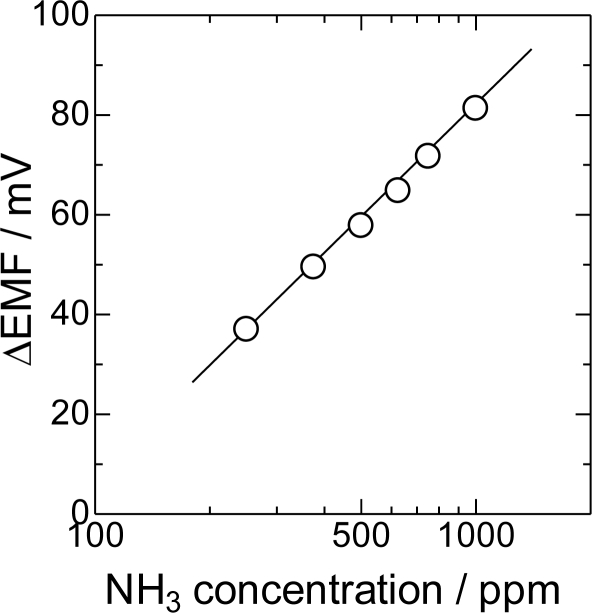
ΔEMF of Bi_2_O_3_(850) electrode as a function of NH_3_ concentration at 600 °C.

The sensing performance of Bi_2_O_3_(850) as an ammonia sensor was investigated. The response of the Bi_2_O_3_(850) electrode was examined by stepwise change in the NH_3_ concentration up to 1,000 ppm. The measured ΔEMF was plotted as a function of NH_3_ concentration in Figure 6. The sensor characteristic was semi-logarithmic to the NH_3_ concentration. This means that the sensor has a high sensitivity, even at lower ammonia concentrations.

The mixed potential mechanism was evaluated for the Bi_2_O_3_(850) electrode by modified polarization curves [25–27] in 10% O_2_ and in 500 ppm NH_3_ at 600 °C, in the same manner proposed by Miura [25]. The intersection of the polarization curves was observed at the same ΔEMF value in the presence of the same concentrations of NH_3_ and O_2_. When the following Equation (1) at the cathode and Equation (2) at the anode proceed at an equal rate, an electrode potential shows the mixed potential:

(1) $$1/2\;O_{2}+2\;e^{-}\to O^{2-}$$

(2) $$2/3\;{\text{NH}}_{3}+O^{2-}\to 1/3\;N_{2}+H_{2}O+2\;e^{-}$$

**Figure 7.**
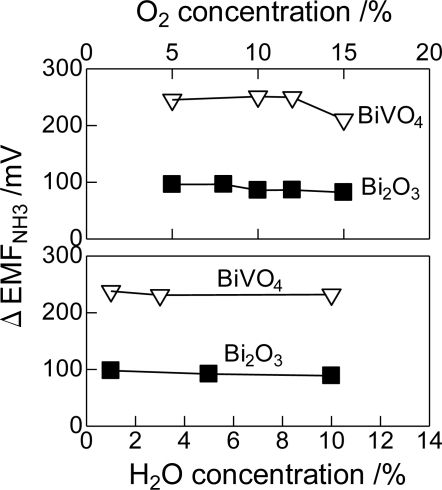
Influence of O_2_ and water vapor on the response of the Bi_2_O_3_(850) and BiVO_4_(750) electrodes to 500 ppm NH_3_ at 600 °C.

It is important to examine the effect of interfering gaseous species on the response of the sensor. Figure 7 shows the influence of the concentration of O_2_ and water vapor on the responses of the Bi_2_O_3_(850) electrode to 500 ppm NH_3_. It should be noted that a different lot of the Bi_2_O_3_(850) sensor was used in this section, and the sensing responses were compared with those of BiVO_4_(750) electrode, which is known as one of the excellent electrode for NH_3_ sensing [39], prepared by the same manner reported by Wang *et al*. [39]. The concentration of water vapor was varied from 1 to 10%. The responses of both the sensors were not much affected by water vapor. While, the higher concentration of O_2_ slightly decreased the ΔEMF vale of the Bi_2_O_3_(850) electrode, though the ΔEMF of the BiVO_4_(750) electrode significantly decreased when the O_2_ concentration is 15%. The Bi_2_O_3_(850) electrode is less interfered by the concentration of O_2_ than the BiVO_4_(750) electrode.

## 4. Conclusions

The effect of calcination temperature and acid-base properties of metal oxides used as a sensing material for an ammonia sensor have been investigated. The sensor electrode was prepared by the two-chamber cell constructed from a YSZ solid electrolyte, on which one side is covered by a Pt electrode and another side is covered with a mixture of Au and a metal oxide thick film as a sensing material. When MoO_3_, Bi_2_O_3_ and V_2_O_5_, having melting points below 820 °C, are used as the sensing material, the sensors exhibited very high cross-sensitivity to NH_3_. The use of WO_3,_ Nb_2_O_5_ and MgO having melting points above 1,000 °C as sensing materials was not effective. The good spreading of the metal oxide on the sensing electrode was suggested to be one of the important factors. The sensing selectivity to ammonia was in the order of MoO_3_ > Bi_2_O_3_ > V_2_O_5_, which was in agreement with the corresponding acidity of the metal oxides. It was clarified that the acidity of metal oxides is the determining factor for the selective sensing of NH_3_.

## Figures and Tables

**Table 1.** Summary of ΔEMF to 500 ppm of NH_3_ and C_3_H_6_ at 600 °C on the sensor electrode covered with mixtures of Au and various metal oxides.

**Electrode**	**m.p. of MOx**	**Calcination/°C**	**ΔEMF_NH3_**	**ΔEMF_C3H6_**	**ΔEMF_NH3_/ΔEMF_C3H6_**
MnO_2_(700)	535	700	176	19.6	9.0
V_2_O_5_(700)	690	700	188	33.2	5.7
MoO_3_(800)	795	800	336	9.5	35.5
Bi_2_O_3_(850)	820	850	68.2	3.6	18.9
WO_3_(1000)	1,470	1,000	50.9	30.5	1.7
Nb_2_O_5_(1000)	1,520	1,000	224	173	1.3
MgO(1000)	2,800	1,000	190	146	1.3
Au only	(1,064)	1,000	89.4	71.3	1.3
